# Imidazothiazole-based potent inhibitors of V600E-B-RAF kinase with promising anti-melanoma activity: biological and computational studies

**DOI:** 10.1080/14756366.2020.1819260

**Published:** 2020-09-23

**Authors:** Hanan S. Anbar, Mohammed I. El-Gamal, Hamadeh Tarazi, Bong S. Lee, Hong R. Jeon, Dow Kwon, Chang-Hyun Oh

**Affiliations:** a Department of Clinical Pharmacy and Pharmacotherapeutics, Dubai Pharmacy College for Girls, Dubai, United Arab Emirates; b Department of Medicinal Chemistry, College of Pharmacy, University of Sharjah, Sharjah, United Arab Emirates; c Sharjah Institute for Medical Research, University of Sharjah, Sharjah, United Arab Emirates; d Department of Medicinal Chemistry, Faculty of Pharmacy, University of Mansoura, Mansoura, Egypt; e CTC SCIENCE, Hwaseong, Gyeonggi-do, Republic of Korea; f CTCBIO Inc., Hwaseong, Gyeonggi-do, Republic of Korea; g Center for Biomaterials, Korea Institute of Science & Technology (KIST), Seoul, Republic of Korea, Seoul;; h Department of Biomolecular Science, University of Science & Technology (UST), Daejeon, Republic of Korea

**Keywords:** Apoptosis, imidazothiazole, melanoma, modelling, V600E-B-RAF

## Abstract

A series of imidazothiazole derivatives possessing potential activity against melanoma cells were investigated for molecular mechanism of action. The target compounds were tested against V600E-B-RAF and RAF1 kinases. Compound **1zb** is the most potent against both kinases with IC_50_ values 0.978 and 8.2 nM, respectively. It showed relative selectivity against V600E mutant B-RAF kinase. Compound **1zb** was also tested against four melanoma cell lines and exerted superior potency (IC_50_ 0.18-0.59 µM) compared to the reference standard drug, sorafenib (IC_50_ 1.95-5.45 µM). Compound **1zb** demonstrated also prominent selectivity towards melanoma cells than normal skin cells. It was further tested in whole-cell kinase assay and showed in-cell V600E-B-RAF kinase inhibition with IC_50_ of 0.19 µM. Compound **1zb** induces apoptosis not necrosis in the most sensitive melanoma cell line, UACC-62. Furthermore, molecular dynamic and 3D-QSAR studies were done to investigate the binding mode and understand the pharmacophoric features of this series of compounds.

## Graphical Abstract

**Figure:**
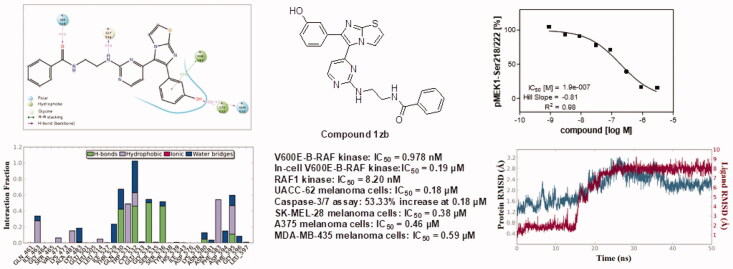

## 1. Introduction

Melanoma is malignant tumour of melanocytes that are present in the skin and the eye. People with fair skin are more prone to melanoma than dark-coloured skin individuals. In addition, blue, green, or hazel-colored eyes are at higher risk to melanoma than dark-coloured eyes^1^. Excessive exposure to sun light and family history of melanoma are also among the risk factors of melanoma. The World Health Organisation (WHO) indicates about 132,000 new melanoma skin cancer cases are diagnosed annually worldwide. Moreover, the American Cancer Society estimates diagnosis of more than 100,000 melanoma cases in USA only in 2020^2^. Much research efforts are required to develop more efficient and less toxic anti-melanoma drugs.

**Figure 1.**
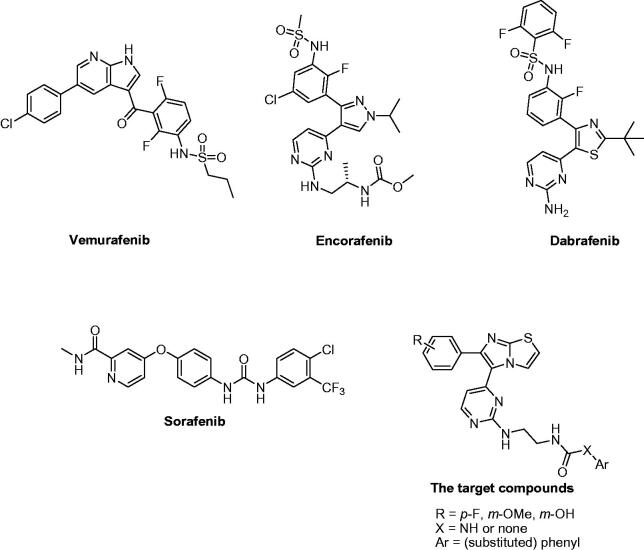
Structures of vemurafenib, encorafenib, dabrafenib, sorafenib, and the target compounds.

RAS-RAF-MEK-ERK pathway is among the potential targets for melanoma treatment. V600E mutated B-RAF is over-activated and over-expressed in several melanoma patients^3^^,^^4^. Inhibitors of V600E-B-RAF and RAF1 (C-RAF) kinases have been reported as antiproliferative agents against melanoma. Among which, vemurafenib^5^, encorafenib^6^, and dabrafenib^7^^,^^8^ (Figure 1) have been marketed as RAF-inhibitory anti-melanoma therapies. Several clinical trials are currently running for these drugs either alone or in combination with other drugs. In addition, sorafenib (Figure 1) is one of the potent inhibitors of RAF kinases that was utilised as a reference standard in our cell-based assay^9^. Moreover, several imidazothiazole derivatives were reported as V600E-B-RAF or RAF1 kinase inhibitors^10–12^.

We have previously reported a series imidazothiazole derivatives as potential antiproliferative agents against melanoma^13–15^. In our previous reports, we did not investigate their molecular mechanism of action. In the present study, we tested their molecular mechanism of action against kinases in cell-free and whole-cell assays. The most potent compound was further tested against melanoma and normal skin cells to investigate the selectivity indexes. The ability of the most potent compound to induce apoptosis and necrosis were also tested. Moreover, different computational studies such as molecular dynamic simulation and QSAR were carried out.

## 2. Experimental

### 2.1. Synthesis of the target compounds

The methods are reported in our previously published articles^13–15^.

### 2.2. Cell-free kinase profiling

In a final volume of 25 µL, the tested kinase (5–10 mU) was incubated with 25 mM Tris pH 7.5, 0.02 mM EDTA, 0.66 mg/mL myelin basic protein, 10 mM magnesium acetate and [γ^33^P-ATP] (specific activity approx. 500 cpm/pmol, concentration as required). The reaction was initiated by the addition of the Mg-ATP mix. After incubation for 40 min at room temperature, the reaction was stopped by the addition of 5 µL of a 3% phosphoric acid solution. 10 µL of the reaction was then spotted onto a P30 filtermat and washed three times for 5 min in 75 mM phosphoric acid and once in methanol before drying and scintillation counting.

### 2.3. In-cell kinase assay

In the cellular V600E-B-RAF phosphorylation assay the murine embryonal fibroblast cell line (MEF) was used, which expresses exogenously introduced constitutively active human V600E-B-RAF fused to the *N*-terminus of a modified oestrogen receptor (ER). In this construct, V600E-B-RAF is only active in the presence of the oestrogen-analogue hydroxytamoxifen. hydroxytamoxifen treatment results in B-RAF-VE600E kinase activation and subsequent phosphorylation of the direct V600E-B-RAF substrate MEK1 at Ser218/222. MEF-V600E-B-RAF:ER cells were plated in Dulbecco’s modified eagle medium (DMEM) supplemented with 10% FCS in multiwell cell culture plates. Compound incubation (90 min at 37 °C) was done in serum-free medium. Cells treated with 10 µM sorafenib were used as low control. For stimulation, cells were treated with 1 µM hydroxytamoxifen for 1 h at room temperature. After cell lysis, quantification of MEK1 phosphorylation was assessed in 96-well plates *via* sandwich-ELISA using a MEK1 specific capture antibody and a phospho-MEK1-Ser218/222 specific detection antibody.

### 2.4. MTT assay

The melanoma and normal skin cell lines were maintained in DMEM including 10% foetal bovine serum (FBS) and 1% penicillin/streptomycin in a humid atmosphere containing 5% carbon dioxide at 37 °C. The cells were taken from culture substrate with 0.05% trypsin-0.02% EDTA and plated at a density of 5 × 10^3^ cells/well in 96-well plates followed by incubation at 37 °C for 24 h in a humid atmosphere with 5% carbon dioxide CO_2_ before treatment with various concentrations (3-fold serial dilution starting with 10 µM, 10 points) of the test compound **1zb** and sorafenib. The cells were incubated for 48 h following treatment with the test compounds. The cellular viability was assessed by the conventional 3–(4,5-dimethylthiazol-2-yl)-2,5-diphenyltetrazolium bromide (MTT) reduction assay. MTT assays were carried out with CellTiter 96® (Promega) according to the manufacturer’s instructions. The absorbance at 590 nm was recorded using EnVision 2103 (Perkin Elmer; Boston, MA, USA). The IC_50_ values were calculated using GraphPad Prism 4.0 software. Triplicate testing was performed.

### 2.5. Caspase-3/7 and LDH release assays

They were performed following the protocols reported in our previously published article^16^.

### 2.6. Computational studies

#### 2.6.1. Data set

ChemDraw Ultra^®^ software (http://www.cambridgesoft.com) was used for drawing the chemical structures, then the low energy conformations were achieved by MOPAC2012 program (MOPAC2012, J. J. P. Stewart, Stewart Computational Chemistry, version 15.321 web: http://OpenMOPAC.net) embedded within VEGA-ZZ^®^ ^17^^,^^18^ utilising the Austin Model-1 (AM1) semiemperical force-field for accurate energy minimisation.

#### 2.6.2. Docking and molecular dynamic simulation

As a prerequisite for molecular dynamic simulation, docking experiments were carried-out using the program AutoDock Vina^19^. The x-ray crystal structures of V600E-B-RAF kinase (PDB-ID: 3IDP) and RAF1 kinase (PDB-ID: 3OMV) were retrieved from Protein Data Bank (http://www.rcsb.org/pdb/). Bound inhibitors and crystalline water molecules were extracted from the initial structures. Autodock MGL Tools were used for the addition of polar hydrogens and charges. Compound **1zb** was treated using the same procedure. Spacing of 1.0 Å between the grid points was used to establish grid boxes covering the active site of the studied macromolecules, centred towards the coordinates of 5.29 (x), 24.13 (y), 32.82 (z) for V600E-B-RAF and towards the coordinates of 28.61 (x), 39.19 (y), 39.09 (z) for RAF1 kinase. Exhaustiveness was set to 12, while number of poses was set to 10.

Molecular dynamic simulations for the compound **1zb** with respect to V600E-B-RAF and RAF1 were started from the earlier docked complexes. Desmond software^®^ (Desmond Molecular Dynamics System, version3.8, D. E. Shaw Research, New York, NY, 2014) embedded within Maestro interface (Schrödinger Release 2014–2: Maestro, version 9.8, Schrödinger, LLC, New York, NY, 2014) was used to conduct all-atoms molecular dynamic simulations employing OPLS_2005 force field parameters. Each complex was subjected to the same dynamic protocol, in which; TIP3P explicit water molecules as solvent model within an orthorhombic periodic boundary box of the size 10 Å^3^ were used to solvate the protein-ligand complex, then, system neutralisation was accomplished by adding appropriate counter-ions followed by adding 0.15 M of salt ions. Prior to the actual dynamic run, system relaxation was achieved by performing a series of short (2000 iterations) restrained and non-restrained solute minimizations steps followed by short 12 ps simulation steps using NVT and NPT ensembles. 50 ns production run was carried out using the NPT ensemble class integrating the equation of motion every 2 fs and setting the temperature and pressure to 300°K and 1 atmosphere, respectively. Short-range interactions cut-off was set to 9 Å and the long-range electrostatic interactions were calculated employing the particle mesh Ewald (PME) method. Results were visualised within maestro environment and analysed using Desmond interaction diagram panel.

#### 2.6.3. Flap® 3D-QSAR model construction

The 3D-QSAR models for the RAF1 and V600E-B-RAF active compounds were constructed employing the program Flap^®^ (fingerprint for ligands and proteins)^20^^,^^21^. The program calculates fingerprints, which are derived from the GRID Molecular Interaction Fields (MIFs) and are characterised as quadruplets of pharmacophoric features. Model construction was initiated by building a compound database for each enzyme. For the RAF1 enzyme, 9 active compounds with their available *p*IC_50_ values were used for this purpose, whereas 22 active compounds with their corresponding *p*IC_50_ values (Table 6) were used for the V600E-B-RAF database creation. Next, the protonation states for each molecule at *p*H 7.4 were calculated using an integrated Flap® tool called MoKa^®^^22^. Subsequently, for each molecule, a maximum of 50 conformers were generated with an RMSD value of 0.3 Å. Ligand-based alignment was achieved employing the most active compound **1zb** in its low energy conformation as a template. Later, for each database, a partial least square (PLS) model was constructed employing the GRID-probes; H (shape), DRY (hydrophobic), O (H-bond acceptor) and N1 (H-bond donor). Model validation was accomplished *via* leave-one-out cross validation and the optimal latent-variables for each model were determined by investigating the R^2^ *vs* Q^2^ plot.

**Table 6.** The calculated VolSurf+^®^ descriptors employed for the QSAR models construction.

Compound	*p*IC_50_ (V600E- B-RAF)	*p*IC_50_ (RAF1)	*IW1*	*IW2*	*FLEX*	*PSA*	*ACDODO*	*DD8*
**1b**	6.611	–	0.031	0.081	3.740	122.660	12.117	0.250
**1e**	6.842	–	0.045	0.112	2.772	99.200	7.005	1.500
**1f**	7.077	–	0.049	0.126	2.834	99.200	7.135	0.125
**1h**	6.719	–	0.040	0.092	3.433	122.060	7.896	0.000
**1i**	6.848	–	0.024	0.054	3.577	122.060	7.773	0.375
**1j**	7.107	–	0.033	0.077	3.048	119.430	14.874	0.875
**1m**	7.083	–	0.012	0.028	4.289	119.940	6.937	0.625
**1n**	6.921	–	0.011	0.021	4.106	119.840	8.935	2.875
**1r**	7.951	7.341	0.033	0.087	3.748	126.060	26.359	0.000
**1s**	7.735	7.304	0.017	0.056	4.217	131.160	25.881	1.750
**1t**	6.712	–	0.046	0.118	4.984	136.260	23.780	0.250
**1u**	7.346	–	0.027	0.078	3.353	105.230	6.998	0.250
**1v**	7.000	–	0.024	0.066	3.592	116.660	6.230	0.500
**1w**	6.917	–	0.037	0.095	4.087	110.330	5.902	0.250
**1z**	6.618	–	0.025	0.065	4.241	125.870	6.422	0.250
**1zb**	9.010	8.086	0.027	0.075	3.237	114.030	23.318	1.000
**1zc**	8.721	7.979	0.018	0.047	3.510	134.260	25.265	0.000
**1zd**	8.469	7.777	0.013	0.044	3.874	119.130	16.949	0.000
**1ze**	7.541	6.330	0.048	0.108	3.975	124.230	25.509	1.625
**1zf**	7.654	6.821	0.021	0.061	4.241	134.770	23.943	0.250
**1zg**	7.801	6.801	0.020	0.045	4.350	134.670	28.806	2.750
**1zh**	8.131	7.206	0.018	0.041	4.085	134.670	23.955	0.500

#### 2.6.4. Volsurf+^®^ QSAR model construction

Two additional QSAR models were constructed; one for the RAF1 enzyme, and another one for the V600E-B-RAF. In each case, the molecular descriptors for the studied compounds were calculated employing the software VolSurf+^®^ ^23^^,^^24^. The software calculates a set of 1D-3D molecular descriptors (*ca.* 128) of different classes including molecular size/shape, hydrophilic/hydrophobic regions quantification, INTEraction enerGY (INTEGY moments), capacity factors, amphiphilic moments, hydro-lipo balance, molecular diffusivity, LogP, LogD, *p*H-dependent solubility, molecular flexibility, 3D pharmacophoric descriptors, *etc*. During model development, the calculated descriptors were recruited as independent variables, whereas the bioactivity values (*p*IC_50_) for the compounds under study were recruited as the dependent variable. Development and validation of our QSAR models were achieved employing the QSARINS^®^ software^25^^,^^26^. All subsets were investigated for the first two descriptors selection. Then, optimal combinations of descriptors (greater than two) were reached by means of genetic algorithm (GA). In the GA selection phase, the population size, maximum number of generations and mutation rate were set to 800, 3000 and 0.6, respectively. Multiple linear regression (GA-MLR) method was adapted for the final model building. The robustness for each model was assessed employing the *Q*^2^_LOO_ (leave one-out) cross validation procedure.

## 3. Results and discussion

### 3.1. Chemistry

**Scheme 1.**
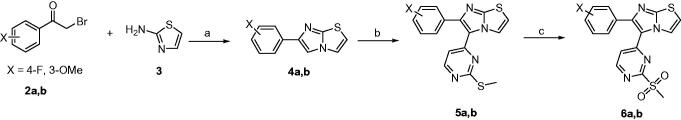
Reagents and conditions: (A) EtOH, reflux, 16 h; (B) 4-iodo-2-(methylthio)pyrimidine, Pd(OAc)_2_, Cs_2_CO_3_, PPh_3_, DMF, 80 °C, 12 h; (C) oxone, MeOH, H_2_O, rt, 16 h.

**Scheme 2.**
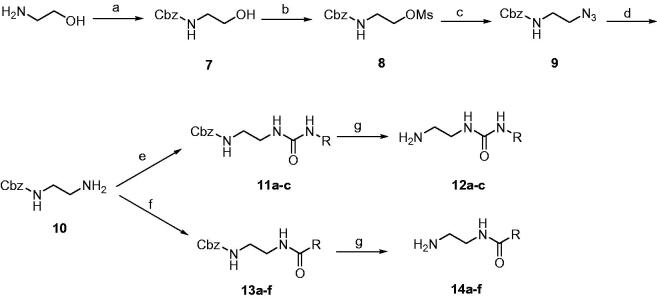
Reagents and conditions: (A) benzyloxycarbonyl chloride, TEA, CH_2_Cl_2_, 0 °C; (B) methanesulfonyl chloride, TEA, CH_2_Cl_2_, 0 °C; (C) NaN_3_, DMSO, 70 °C, 2 h; (D) PPh_3_, MeOH, H_2_O, reflux, 2 h; (E) appropriate isocyanate, THF, rt, 2 h; (F) appropriate carboxylic acid, HOBt, EDCI, TEA, DMF, 80 °C, 8 h; (G) H_2_/Pd-C, MeOH, rt, 1 h.

**Scheme 3.**
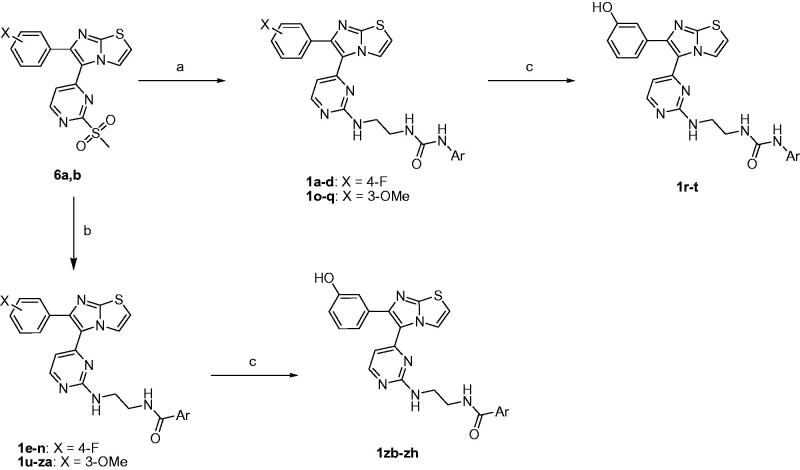
Reagents and conditions: (A) **12a-c**, DIPEA, DMSO, 80 °C, 8 h; (B) **14a-f**, DIPEA, DMSO, 80 °C, 8 h; (C) BBr_3_, CH_2_Cl_2_, –78 °C, 30 min then rt, 1 h.

The target compounds **1a-zh** were prepared utilising the pathways illustrated in Schemes 1–3^13–15^. The synthetic strategy involved preparation of methyl sulphonyl intermediates **6a,b** (Scheme 1) and amine side chain intermediates **13a-c** and **15a-f** (Scheme 2), followed by interaction of them together to get the aminopyrimidinyl final compounds (Scheme 3).

Scheme 1 starts with a cyclisation reaction between 2-aminothiazole (**3**) and bromoacetophenone derivatives (**2a,b**) in refluxing ethanol to get imidazothiazole intermediates **4a,b**^27^. Coupling of compounds **4a,b** with 4-iodo-2-(methylthio)pyrimidine using palladium(II) acetate, caesium carbonate, and triphenylphosphine as catalysts led to formation of diarylimidazothiazole intermediates **5a,b**. Oxidation of the methylthio group of compounds **5a,b** using oxone yielded the corresponding methyl sulphonyl derivatives **6a,b**.

Scheme 2 illustrates the conversion of 2-aminoethanol into the amino reagents possessing arylamide or arylurea moiety. At the beginning, the amino group of 2-aminoethanol was Cbz-protected using benzyloxycarbonyl chloride in presence of triethylamine (TEA)^28^. The hydroxyl group of compound **7** was converted into methanesulfonate using methanesulfonyl chloride and TEA^29^. Compound **8** was heated with sodium azide to replace the OMs group with azido (compound **9**). Reduction of azido compound **9** into the corresponding amino was accomplished using triphenylphosphine in refluxing methanol. The free primary amino group of compound **10** was treated with aryl isocyanates or condensed with carboxylic acids to form the corresponding urea or amide, respectively. The last step of Scheme 2 involves deprotection of Cbz group using hydrogen gas and palladium metal over charcoal.

In Scheme 3, the mesyl intermediates **6a,b** were heated with the amino side chain reagents **12a-c** and **14a-f** in presence of Hunig’s base to produce the target compounds possessing fluoro or methoxy on the benzene ring at position 6 of the imidazothiazole nucleus. Boron tribromide was used to demethylate the methoxy compounds **1o-q** and **1 u-za** to form the corresponding phenolic analogues **1r-t** and **1zb-zh**, respectively. The structures of the target compounds **1a-zh** are drawn in Table 1.

**Table 1.** Structures of the target compounds 1a-zh and their inhibitory effects against RAF1 and V600E-B-RAF kinases. The inhibition percentage results are expressed as means of duplicate assays ± SEM

Compound no.	Structure	One-dose (1 µM) results against RAF1 (% inhibition)	One-dose (1 µM) results against V600E-B-RAF (% inhibition)	IC_50_ value (nM) against RAF1	IC_50_ value (nM) against V600E-B-RAF
1a		54.04% ± 5.07%	67.62% ± 3.61%	ND	ND
1b		63.81% ± 2.51%	73.06 ± 1.72%	ND	245 nM
1c		59.53% ± 2.25%	52.03% ± 3.61%	ND	ND
1d		63.44% ± 1.62%	69.37% ± 0.05%	ND	ND
1e		62.63% ± 0.34%	90.43% ± 0.72%	ND	144 nM
1f		71.39% ± 1.32%	90.99% ± 0.56%	ND	83.8 nM
1g		13.99% ± 2.28%	38.64% ± 0.21%	ND	ND
1h		38.67% ± 5.01%	77.90% ± 0.11%	ND	191 nM
1i		55.46% ± 1.61%	85.45% ± 0.42%	ND	142 nM
1j		69.84% ± 0.52%	91.40% ± 0.48%	ND	78.2 nM
1k		66.14% ± 2.60%	−5.63 ± 3.11%	ND	ND
1l		13.09% ± 0.12%	40.59% ± 1.72%	ND	ND
1m		75.89% ± 1.43%	80.25% ± 0.61%	ND	82.6 nM
1n		75.69% ± 0.67%	84.82% ± 0.42%	ND	120 nM
1o		32.50% ± 1.91%	52.76% ± 3.66%	ND	ND
1p		60.68% ± 0.95%	69.65% ± 0.40%	ND	ND
1q		19.33% ± 2.37%	23.59% ± 1.99%	ND	ND
1r		97.45% ± 0.10%	97.66% ± 0.16%	45.6 nM	11.2 nM
1s		97.28% ± 0.08%	99.54% ± 0.23%	49.7 nM	18.4 nM
1t		67.56% ± 4.79%	87.74% ± 0.38%	ND	194 nM
1u		74.00% ± 0.64%	95.03% ± 0.66%	ND	45.1 nM
1v		59.25% ± 2.53%	90.82% ± 0.25%	ND	100 nM
1w		56.61% ± 2.15%	83.65% ± 0.47%	ND	121 nM
1x		32.14% ± 0.08%	56.21% ± 0.10%	ND	ND
1y		57.47% ± 0.47%	63.23% ± 0.93%	ND	ND
1z		32.12% ± 2.26%	79.41% ± 0.56%	ND	241 nM
1za		51.51% ± 3.74%	71.56% ± 3.43%	ND	ND
1zb		99.39% ± 0.20%	99.48% ± 0.09%	8.2 nM	0.978 nM
1zc		99.32% ± 0.23%	101.07% ± 1.53%	10.5 nM	1.9 nM
1zd		98.71% ± 0.23%	100.24% ± 0.17%	16.7 nM	3.4 nM
1ze		86.02% ± 1.08%	97.81% ± 1.67%	468.0 nM	28.8 nM
1zf		92.71% ± 0.99%	97.19% ± 0.32%	151.0 nM	22.2 nM
1zg		90.78% ± 0.22%	98.97% ± 0.43%	158.0 nM	15.8 nM
1zh		96.60% ± 0.18%	100.66% ± 2.35%	62.2 nM	7.4 nM

### 3.2. Kinase profiling

Based on our previous knowledge of inhibitory effect of imidazothiazole derivatives against V600E-B-RAF and RAF1 ^11^^,^^12^, we decided to start our biological investigation with testing all the 34 final compounds against both kinases. The target compounds were tested at 1 µM concentration, and the compounds that produced promising inhibitory effect, more than 75% inhibition, were further tested in 10-dose mode to measure their IC_50_ values. The inhibition percentage and IC_50_ values are demonstrated in Table 1. The target compounds are generally more active against V600E-B-RAF than RAF1. A comparative computational study was carried out to investigate it.

*Meta*-hydroxyl group on the phenyl ring at position 6 of the imidazothiazole nucleus is more favourable for activity than *meta*-methoxy and *para*-fluoro substituents against both V600E-B-RAF and RAF1. This is emphasised by the activity of OH compounds **1zb-zh** compared with OMe analogues **1 u-za** or F derivatives **1e** and **1j-n**. It is obvious that hydrogen bond donor at the this part of the structure is optimal for stronger affinity with V600E-B-RAF and RAF1 kinases. This was further investigated by computational studies. In addition, amide compounds (**1e**, **1 u**, **1x**, **1zb**, **1zd**, and **1ze**) are more active and more potent than the corresponding urea analogues (**1a**, **1o**, and **1q-t**). It can be concluded that the length, hydrophobicity, and electronic properties of amide spacer are more optimal for activity than those of urea. Furthermore, terminal benzamido moiety with no substitution (e.g. compounds **1 u** and **1zb**) or *ortho*-hydroxyl group (e.g. compounds **1v** and **1zc**) are more favourable for activity than analogues with bulkier substituents. QSAR study was carried out to further investigate this issue. Among all the final compounds, compound **1zb** possessing 3-hydroxyphenyl at position 6 of imidazothiazole and unsubstituted benzamido is the most potent against both V600E-B-RAF and RAF1. It is the only compound that exerted sub-nanomolar IC_50_ value against V600E-B-RAF.

The most potent compound, **1zb**, against V600E-B-RAF and RAF1 was further tested at 1 µM concentration against a 30-kinase panel belonging to different kinase families. The results are illustrated in Table 2. Apart from V600E-B-RAF, it inhibited 73.14% of FLT3 kinase while its inhibitory effect against all the other kinases was less than 50%. The IC_50_ of compound **1zb** against FLT3 was further examined and found to be 838 nM (Table 3). So the compound is 856.85 times more selective towards V600E-B-RAF than FLT3. It is also 8.38 folds more selective against V600E-B-RAF than RAF1. Based on these results, compound **1zb** can be considered as a relatively selective V600E-B-RAF kinase inhibitor.

**Table 2.** Kinase profiling of compound **1zb** (1 µM) against a 30-kinase panel. The results are expressed as mean %inhibition ± SEM

Kinase	% inhibition
ABL	7.57% ± 2.5%
ALK	41.16% ± 1.5%
A-RAF	9.14% ± 1.0%
Aurora-A	49.77% ± 5.5%
B-RAF^wt^	23.37% ± 0.9%
BTK	16.96% ± 0.8%
DMPK	0.84% ± 0.1%
EGFR	19.93% ± 2.0%
ErbB2	2.17% ± 0.9%
ErbB4	13.67% ± 2.8%
FLT3	73.14% ± 2.0%
FMS	12.19% ± 0%
JAK1	−0.26% ± 0%
JAK2	3.76% ± 0.5%
JNK1(α1)	0.76% ± 0.1%
KDR	18.76% ± 1.0%
cKIT	9.63% ± 0%
LCK	12.45% ± 2.5%
MEK1	1.50% ± 0.2%
MEK2	26.60% ± 2.5%
mTOR	7.83% ± 0.3%
PDGFRβ	12.54% ± 1.5%
PI3Kα	5.85% ± 0%
PI3K-C2α	49.99% ± 2.3%
ROS	2.02% ± 0.3%
Src(T341M)	1.57% ± 0.3%
STK33	15.65% ± 0.5%
TRKA	46.72% ± 1.5%
VRK1	3.93% ± 0.5%
V600E-B-RAF	**99.48% ± 0.09%**^a^

^a^Data taken from Table 1.

**Table 3.** IC_50_ values of compound **1zb** against V600E-B-RAF, RAF1, and FLT3 kinases.

Kinase	IC_50_ (nM)
V600E-B-RAF	0.978^a^
RAF1	8.2^a^
FLT3	838

^a^Data taken from Table 1.

### 3.3. Antiproliferative activity against melanoma cell lines

Compound **1zb** was tested against four melanoma cell lines and normal skin epithelial cell line. Its potency was compared with sorafenib as a reference standard as it is a potent RAF kinase inhibitor. The results are illustrated in Table 4. Compound **1zb** is more potent than sorafenib against all the four tested melanoma cell lines. Its IC_50_ values are within sub-micromolar range, while those of sorafenib are in one-digit micromolar scale. The selectivity indexes of compound **1zb** against A375, MDA-MB-435, SK-MEL-28, and UACC-62 melanoma cell lines compared to CCD1106K normal skin cells are 20.13, 15.69, 24.37, and 51.44, respectively. Furthermore, the strong potency of compound **1zb** against the tested melanoma cell lines that harbour V600E-B-RAF^30^ emphasises the postulation that V600E-B-RAF could be at least a part of molecular mechanism(s) of antiproliferative action of compound **1zb**.

**Table 4.** Antiproliferative activity (IC_50_ value, µM) of compound **1zb** and sorafenib against melanoma cell lines and normal skin cells.^a^

	Cell line	1zb	Sorafenib
Melanoma cell lines	A375	0.46 ± 0.03	5.45 ± 0.53
	MDA-MB-435	0.59 ± 0.06	3.16 ± 0.20
	SK-MEL-28	0.38 ± 0.05	2. 67 ± 0.15
	UACC-62	0.18 ± 0.02	1.95 ± 0.08
Normal skin epithelial cell line	CCD1106K	9.26 ± 0.38	11.57 ± 0.46

^a^The results are expressed as means of triplicate assays ± SEM

**Table 5.** Caspase-3/7 and LDH release assay results of compound **1zb** at 0.18 µM concentration over UACC-62 melanoma cells.

Assay	Result (compared to control cells treated with DMSO)
Caspase-3/7	53.33% increase
LDH release	0% increase

### 3.4. In-cell kinase assay

**Figure 2.**
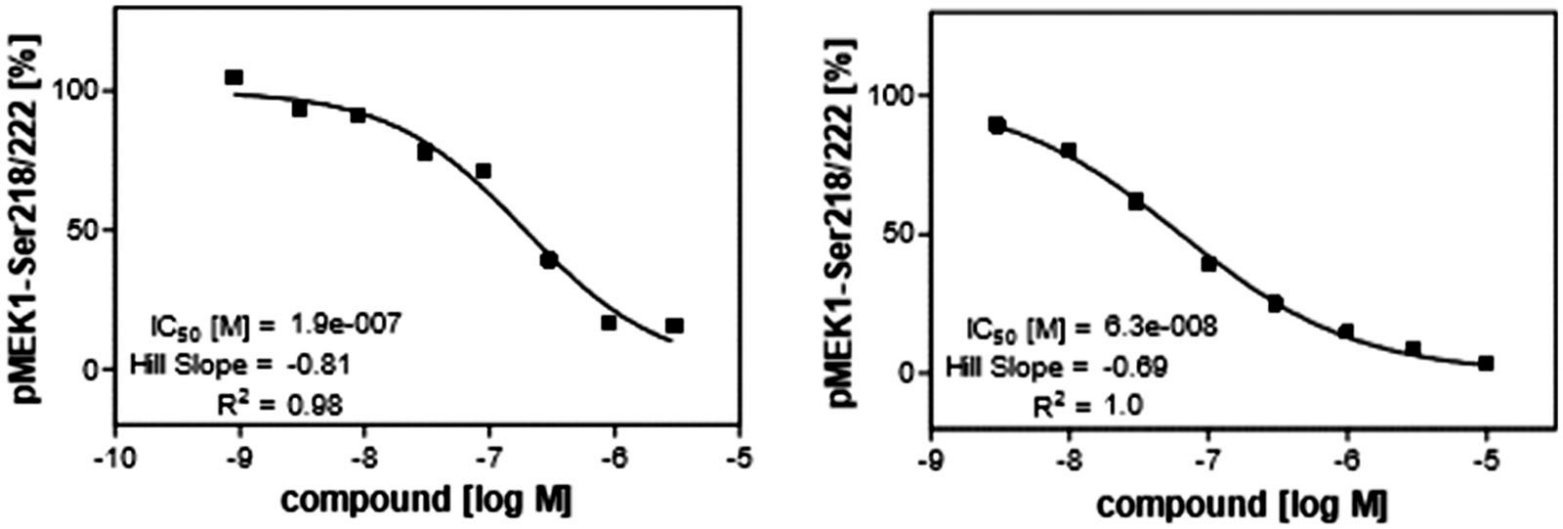
Inhibitory impact of compound **1zb** (left) and sorafenib (right) on the cellular kinase activity of V600E-B-RAF in murine embryonal fibroblast (MEF) cell line.

The next step of our study was investigation of ability of compound **1zb** to inhibit V600E-B-RAF kinase inside the cells after penetrating the cell membrane. It was compared with sorafenib as a reference standard. This assay was done in murine embryonal fibroblast (MEF) cell line and the ability to inhibit V600E-B-RAF was investigated through measuring the concentration of phosphorylated MEK, the downstream protein of V600E-B-RAF. The dose-response curves are illustrated in Figure 2. Compound **1zb** could cross the cell membrane and inhibit V600E-B-RAF with IC_50_ value of 0.19 µM. This concentration is comparable to its IC_50_ value against UACC-62 melanoma cell line (0.18 µM) that has over-expressed V600E-B-RAF^30^. This result can also prove that V600E-B-RAF inhibition could be a molecular mechanism of action against melanoma.

### 3.5. Apoptosis and necrosis assays

As per the results shown in Table 4, UACC-62 is the most sensitive melanoma cell line for compound **1zb**. Its IC_50_ value is 0.18 µM. We decided to investigate whether this concentration could induce apoptosis and/or necrosis in UACC-62 cells. Caspase-3/7 were measured as proof of apoptosis induction while lactate dehydrogenase (LDH) release was measured as an indicator of necrosis^16^. At 0.18 µM concentration, compound **1zb** increased caspase-3/7 release by 53.33% but did not show any significant increase in LDH release (Table 5). This means that compound **1zb** at its IC_50_ concentration can induce apoptosis of UACC-62 melanoma cells but not necrosis. This finding could help us understand its antiproliferative effect in more details.

### 3.6. Computational studies

#### 3.6.1. Molecular dynamic study

**Figure 3.**
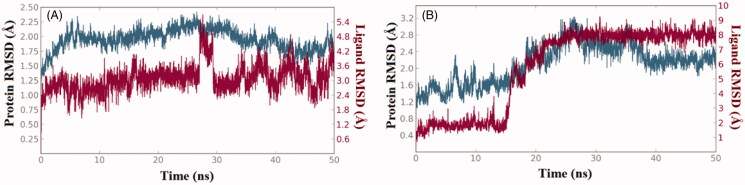
The 50 nanoseconds simulation RMSD values for the proteins and ligands involved; (A) RAF1 kinase Cα-atoms (blue) and compound **1zb** heavy atoms (red), (B) V600E-B-RAF kinase Cα-atoms (blue) and compound **1zb** heavy atoms (red).

The structure-activity relationships presented in Table 1 were further explored *via* computational techniques in an attempt to provide plausible explanation for the bioactivity vibrations existed among our compound series against both enzymes (RAF1 and V600E-B-RAF). Thus, we started our endeavour by performing molecular docking procedure for the most active compound **1zb** against the two enzymes. Then, best docked-complexes achieved were utilised to carry-on a 50 nanosecond (ns) molecular dynamic simulations under explicit hydration environment. In each case, system stability and trajectories were assessed in term of root-mean-squared deviations (RMSD) of protein Cα-atoms as well as the ligand heavy atoms. Figure 3(A,B) illustrate the RMSD values (compared to the starting frame-0) along the simulation time, where changes of the order of 1–3 Å are perfectly acceptable for small, globular proteins. Accordingly, in the case of RAF1 enzyme-ligand complex system equilibration was achieved after 5 ns, while for the V600E-B-RAF; system equilibration was achieved after 18 ns and remained stable during the rest of the simulation time.

**Figure 4.**
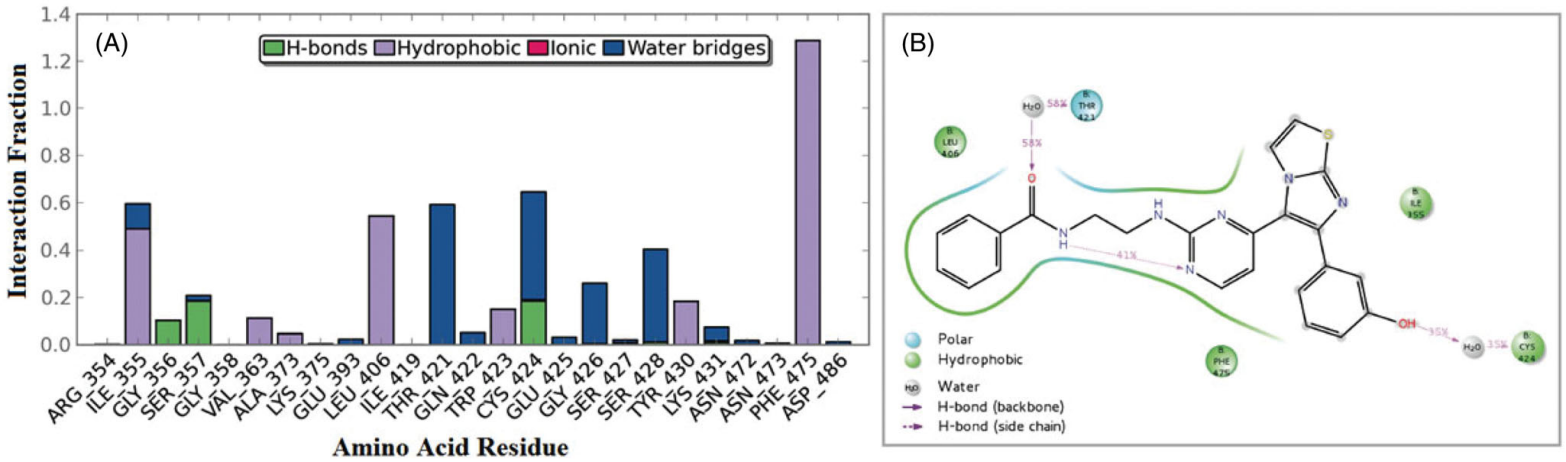
50 nanoseconds simulation interaction diagram panel for **1zb** and RAF1 kinase; (A) Fractions of interaction between **1zb** and RAF1 kinase active site residues, (B) 2 D- interaction diagram of **1zb** within the RAF1 kinase active site.

Interactions of compound **1zb** and the RAF1 kinase revealed a number of important findings. The 3-hydroxyphenyl group (OH) was able to establish hydrogen bonding with the Cys-424 residue for about 65% of the simulation time (direct and water bridge). Additional water bridge interaction was observed between the benzamide carbonyl oxygen (C=O) and the corresponding gatekeeper residue Thr-421 for 58% of the simulation time (Figure 4(A,B)). Furthermore, an interesting intra-molecular hydrogen bonding was observed between the benzamide nitrogen and the pyrimidine nitrogen last for 41% of time, which is indicative for conformational flexibility restriction. Other significant interaction were the hydrophobic interactions with the residues Phe-475, Leu-406, and Ile-355.

**Figure 5.**
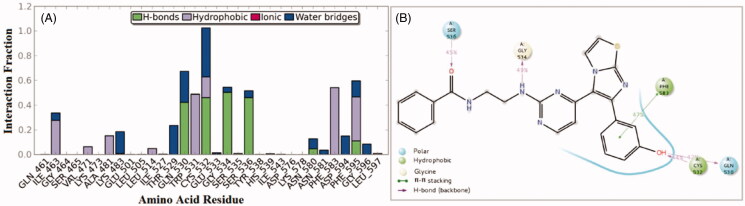
50 nanoseconds simulation interaction diagram panel for **1zb** and V600E-B-RAF; (A) Fractions of interaction between **1zb** and V600E-B-RAF kinase active site residues, (B) 2 D-interaction diagram of **1zb** within the V600E-B-RAF kinase active site.

On the other hand, an analysis of compound **1zb** within the V600E-B-RAF kinase revealed another set of interactions. The 3-hydroxyphenyl group (OH) was able to establish two hydrogen bonds (direct and water-bridged); one with the hinge Cys-532 residue and another one with the Gln-530 residues for about 83% and 65% of the simulation time, respectively. Additional direct H-bond interaction was observed between the benzamide carbonyl oxygen (C=O) and the corresponding residue Ser-536 for about 45% of the simulation time (Figure 5(A,B)). The residue Gly-534 was able to form H-bond with the amino group (NH) next to pyrimidine ring for about 49% of the simulation time. Other hydrophobic interactions were those with the residues Phe-583 and Phe-595.

#### 3.6.2. Flap^®^ 3D-QSAR study

At the next level of our investigation, we were interested in gaining more detailed information about the crucial factors affecting selectivity in each enzyme case, and the contribution of structural variations to the observed potency, thus we decided to conduct a 3D-QSAR analysis employing the program Flap^®^^20^^,^^21^. The program frequently used for building QSAR models based on molecular interaction fields (MIFs) generated through the GRID force-field^31^. Four different GRID-probes were utilised for calculating the final interaction fields including; H-probe for computing the shape constraints on small ligand or protein cavity, DRY-probe for computing favourable hydrophobic interactions, O-probe (carbonyl oxygen) acting as hydrogen bond acceptor (HBA) and N1-probe (amidic NH) acting as hydrogen bond donor (HBD) probe.

**Figure 6.**
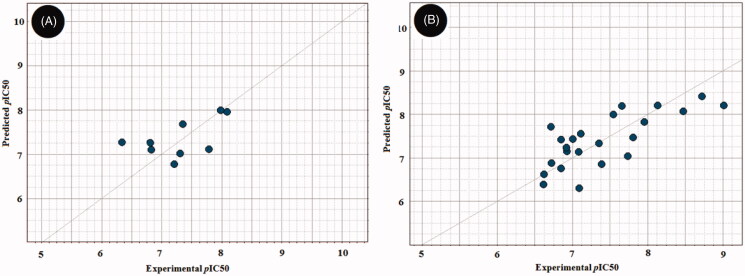
Scatter plots of experimental *vs.* predicted bioactivities (expressed as *p*IC_50_) derived from; (A) RAF1 Flap^®^ 3 D-QSAR model (2-Latent variables, R^2^=0.799), (B) V600E-B-RAF Flap^®^ 3 D-QSAR model (3-Latent variables, R^2^=0.857).

**Figure 7.**
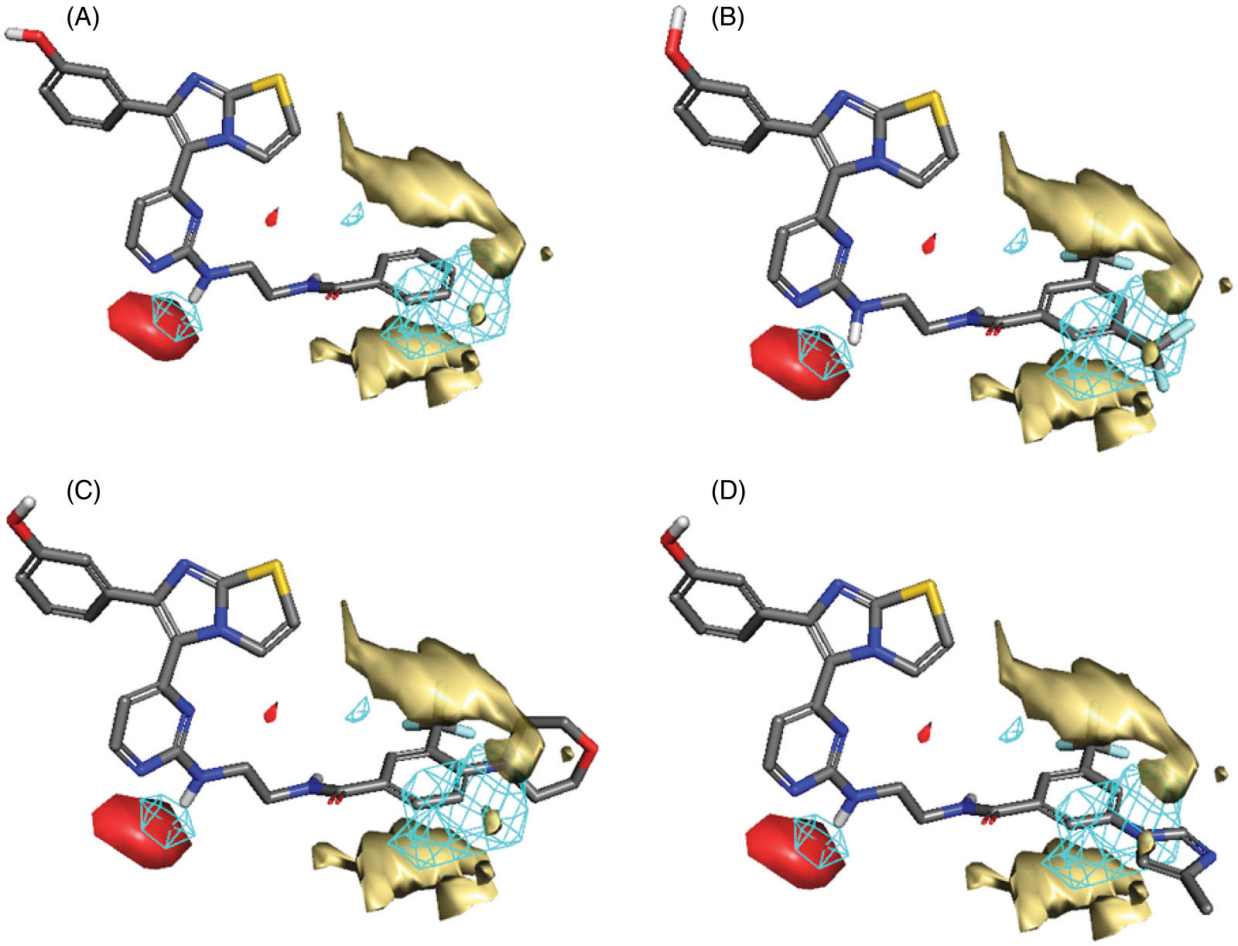
Flap® 3 D-QSAR model generated for the RAF1 compounds; (A) alignment of the most potent compound **1zb**, (B) alignment of compound **1ze**, (C) alignment of compound **1zg**, (D) alignment of compound **1zf**. The GRID molecular interaction fields are shown as; cyan (Shape), yellow (hydrophobic) and red (HBA).

In the case of RAF1 compounds, the best developed model was a two-Latent variables model with an R^2^ of 0.799 (Figure 6(A)) consisted of three interaction fields encoding shape, hydrophobic and HBA regions. Alignment of the most potent compound (**1zb**, IC_50_ = 8.2 nM) to the model (Figure 7(A)) revealed a good match between its structural components and the given fields, where the amino group (NH) next to pyrimidine ring is matching the HBA field (red) and the benzamide ring is matching well with the hydrophobic field (yellow) and there is no shape violation observed at the benzamide ring. On the other hand, the least potent compounds **1ze**, **1zg** and **1zf** exhibited the same structural components match except for the shape field. The trifluoromethyl groups on the benzamide ring of compound **1ze** clearly violating the shape constrains (Figure 7(B)). The same phenomenon was observed for the 4-morpholino substitution of **1zg** (Figure 7(C)) and the 5-methylimidazole ring substitution of **1zf** (Figure 7(D)). Accordingly, it seems that any benzamide substitution violating of the shape constraints would have negative impact on the RAF1 bioactivity and only small compacted substituents could be tolerated.

**Figure 8.**
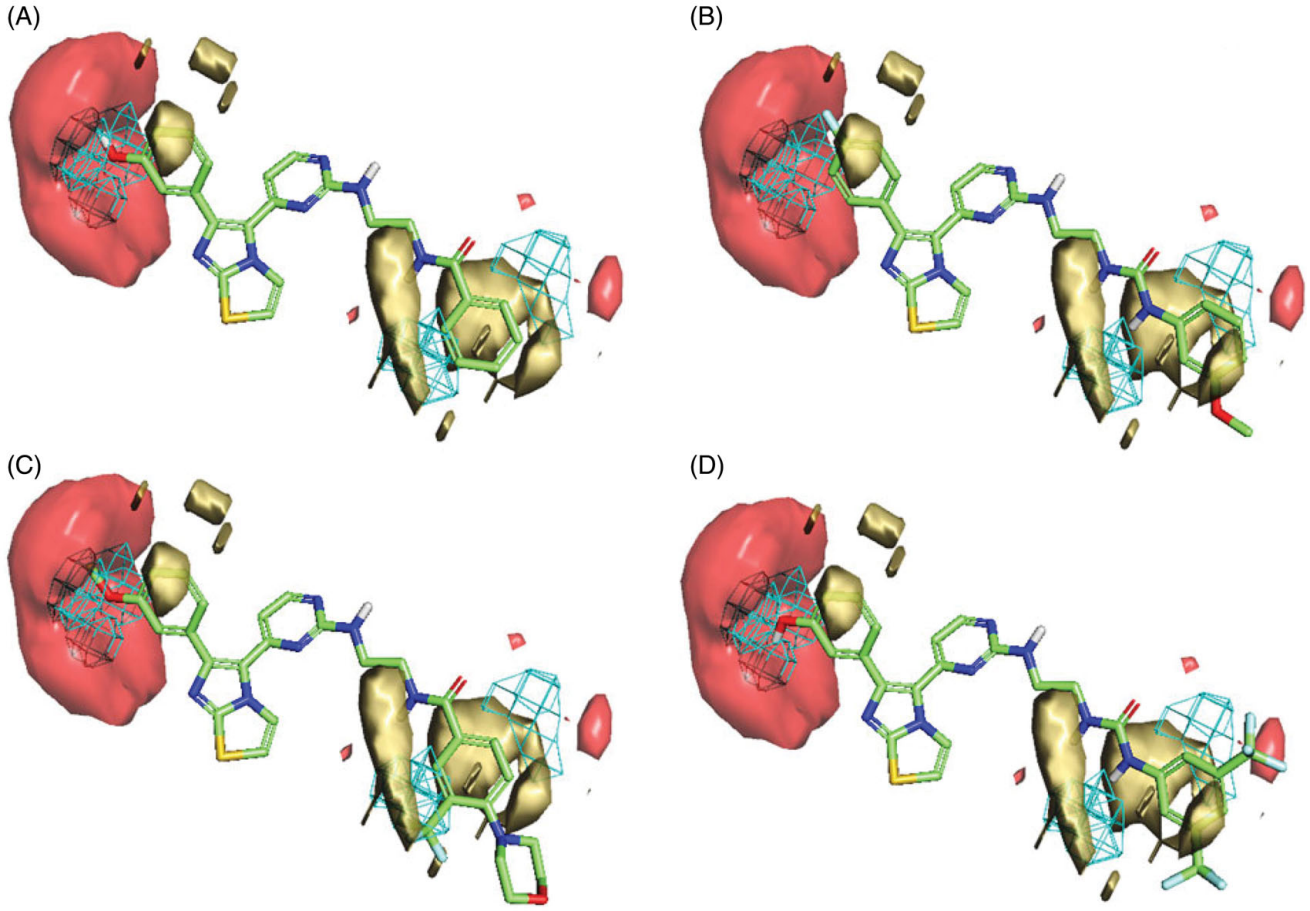
Flap® 3 D-QSAR model generated for the V600E-B-RAF compounds; (A) alignment of the most potent compound **1zb**, (B) alignment of compound **1b**, (C) alignment of compound **1z**, (D) alignment of compound **1t**. The GRID molecular interaction fields are shown as; cyan (Shape), yellow (hydrophobic) and red (HBA).

In the case of V600E-B-RAF compounds, the best developed model was a three-Latent variables model with an R^2^ of 0.857 (Figure 6(B)) consisted of three interaction fields encoding shape, hydrophobic and HBA regions. Again, alignment of the most potent compound (**1*zb***, IC_50_ = 0.978 nM) to the model (Figure 8(A)) revealed a good match between its structural components and the given fields, where the 3-hydroxyphenyl group (OH) is matching the HBA (red) and the shape (blue mesh) fields. The benzamide ring is matching well with the hydrophobic field (yellow) and there is no shape violation observed at the benzamide ring. The least potent compound (**1 b**, IC_50_ = 245 nM) suffers from many drawbacks as observed in Figure 8(B). The 4-fluorophenyl group deviates from the perfect shape match and provide no complementarity with the HBA field. Additionally, the presence of urea linker appeared inferior leading to misalignment of the phenylurea ring from the perfect hydrophobic ring match. In compound **1z** (Figure 8(C)), the presence of 3-methoxyphenyl (HBA) in front of HBA field and the shape constraints violation by the benzamide ring substitutions (3-trifluoromethyl and 4-morpholino) appeared inferior to the bioactivity. The lower potency observed for compound **1t** (Figure 8(D)) could be attributed to the presence of urea linker and the phenylurea ring misalignment. In light of this information, it seems that the presence of small HBD group such as (OH) at *meta*-position of the phenyl ring is highly recommended, and the amide linker is more preferred over the urea linker. Furthermore, small compacted substituents at the *ortho* and *meta* positions of the benzamide ring are of more positive impact for the bioactivity.

#### 3.6.3. Volsurf+^®^ QSAR study

**Figure 9.**
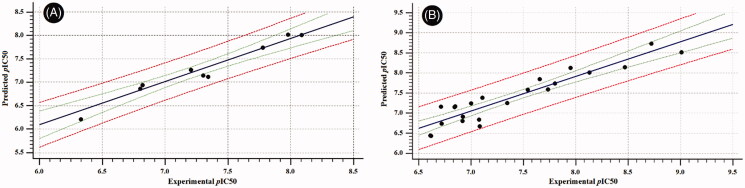
Experimental *versus* predicted bioactivities scatter graphs derived from (A) The developed QSAR equation for the RAF1 kinase. (B) The developed QSAR equation for the V600E-B-RAF kinase. In each graph, solid line is the regression line, green dashed-lines representing the 95% confidence limit, while the red dashed-lines representing the 95% prediction limits.

Later, we turned our focus towards developing QSAR models for the RAF1 and V600E-B-RAF enzymes based on multiple linear regressions (MLR) analysis of molecular descriptors (*ca.* 128) generated by VolSurf+^®^ software^23^^,^^24^ so that to gain more insight of structural trends responsible for activity discrimination. Two statistically significant models were achieved and found to fit well with the experimental biodata (Figures 9(A,B)) as indicated by their corresponding Fischer’s value (F), squared correlation coefficients (R^2^), and the standard errors of estimate (s).

The best developed model capable of describing the inhibitory pattern exerted by our compounds against the C-RAF enzyme is illustrated by the following equation;

pIC50=−35.647×IW1−1.367×FLEX+13.505

***n*** = 9, ***F***-Statistic = 102.77, ***R^2^*** = 0.972, ***Q^2^***_LOO_ = 0.946, ***s*** = 0.114 where in this model; ***IW1*** is the INTEGY (**INTE**raction ener**GY**) moment presented as vector pointing from centre of mass to the centre of the hydrophilic region, Low INTEGY descriptors such as IW1 indicates the hydrophilic moieties are either close to the centre of mass or they balance at an opposite ends of the molecule. ***Flex*** is the maximum flexibility of the molecule estimated after the random generation of 50 conformers; such descriptor is calculated as the logarithmic averaged differences between maximum and minimum distances of atom “*i*” in a selected conformer.

The two descriptors found to negatively influence the bioactivity as demonstrated by their corresponding negative coefficients, hence, for more improved RAF1 inhibitory activity, the model suggested lesser molecular flexibility which is in agreement with the intra-molecular HB observed during molecular dynamic simulation. Furthermore, distribution of hydrophilic moieties towards the terminal ends of the structure would greatly improve the activity (Table 6).

On the other hand, the best developed model for the V600E-B-RAF enzyme is illustrated by the following equation.

pIC50=0.111×ACDODO−17.193×IW2−0.339×DD8−0.062×PSA+14.780

***n*** = 22, ***F***-Statistic = 41.33, ***R^2^*** = 0.907, ***Q^2^***_LOO_ = 0.852, ***s*** = 0.238 where, ***ACDODO*** is a 3D-pharmacophoric descriptor based on the triplets of pharmacophoric points, where it represents an Acceptor-Donor-Donor triplet. ***IW2*** is the INTEGY (**INTE**raction ener**GY**) moment presented as vector pointing from centre of mass to the centre of the hydrophilic region, Low INTEGY descriptors such as IW2 indicates the hydrophilic moieties are either close to the centre of mass or they balance at an opposite ends of the molecule. ***DD8*** is the difference between the maximum hydrophobic volumes (obtained upon variation of ligand conformations) and the hydrophobic volume (D8) of the imported 3D structure calculated at the 8 levels of energy. Furthermore, the hydrophobic volume (D8) is the molecular envelope generating attractive hydrophobic interactions calculated using the DRY-probe at an energy range of −1.6 kcal/mol. ***PSA*** is the Polar Surface Area calculated *via* the sum of polar region contributions.

Among the emerged descriptors, three were found to negatively influence activity (IW2, DD8 and PSA), while one descriptor (ACDODO) found to have positive impact. Thus, the model suggested the needed requirements for improved V600E-B-RAF bioactivity as follow; 1) the presence of three pharmacophoric points (Acceptor-Donor-Donor) is greatly preferred, which is consistent with the dynamic profile discussed earlier, 2) the proper positioning of the small hydrophilic moieties towards the terminal rings is highly advised and 3) the proper hydrophilic-lipophilic balance is highly desirable (Table 6).

## 4. Conclusion

In this study, we studied the molecular mechanism of action of a promising series of imidazothiazole derivatives that are potent against melanoma cells. The target compounds were tested against V600E-B-RAF and RAF1 kinases. The structure-activity relationship study revealed the importance of the following structural moieties for activity; *meta*-hydroxyphenyl moiety at position 6 of the imidazothiazole nucleus, amide linker, and terminal benzene ring attached to the amide spacer either unsubstituted or substituted with a small group. The most promising compound that possesses all these pharmacophoric features is compound **1zb**. It showed extremely high potency and relative selectivity towards V600E-B-RAF. Moreover, compound **1zb** exerted high potency against different melanoma cell lines with sub-micromolar IC_50_ values. It also showed high selectivity towards melanoma cells than normal skin cells. Furthermore, it could penetrate the cell membrane and inhibit V600E-B-RAF kinase with IC_50_ value of 0.19 µM that is comparable to its IC_50_ value against UACC-62 melanoma cells (0.18 µM). At 0.18 µM concentration, it induced caspase-3/7 release that lead to apoptosis. These promising results of compound **1zb** especially makes it a promising drug candidate for further consideration and optimisation to end up with a new promising anti-melanoma agent.

## References

[1] https://www.who.int/news-room/q-a-detail/ultraviolet-(uv)-radiation-and-skin-cancer. Accessed on Aug 3, 2020.

[2] https://www.cancer.org/cancer/melanoma-skin-cancer/about/key-statistics.html. Accessed on Aug. 3^rd^, 2020.

[3] Rajagopalan H, Bardelli A, Lengauer C, et al. Tumorigenesis: RAF/RAS oncogenes and mismatch-repair status. Nature 2002;418:934. 12198537 10.1038/418934a

[4] Abdel-Maksoud M, El-Gamal M, Gamal El-Din M, et al. Design, synthesis, in vitro anticancer evaluation, kinase inhibitory effects, and pharmacokinetic profile of new 1,3,4-triarylpyrazole derivatives possessing terminal sulfonamide moiety. J Enzyme Inhib Med Chem 2019;34:97–109. 30362383 10.1080/14756366.2018.1530225 PMC6211260

[5] Bollag G, Hirth P, Tsai J, et al. Clinical efficacy of a RAF inhibitor needs broad target blockade in BRAF-mutant melanoma. Nature 2010;467:596–9. 20823850 10.1038/nature09454 PMC2948082

[6] Honndorf VS, Coudevylle N, Laufer S, et al. Dynamics in the p38alpha MAP kinase-SB203580 complex observed by liquid-state NMR spectroscopy. Angew Chem Int Ed Engl 2008;47:3548–51. 18389508 10.1002/anie.200705614

[7] Rheault TR, Stellwagen JC, Adjabeng GM, et al. Discovery of Dabrafenib: a selective inhibitor of Raf kinases with antitumor activity against B-Raf-driven tumors. ACS Med Chem Lett 2013;4:358–62. 24900673 10.1021/ml4000063 PMC4027516

[8] Karoulia Z, Gavathiotis E, Poulikakos PI. New perspectives for targeting RAF kinase in human cancer. Nat. Rev. Cancer 2017;17:676–91. 28984291 10.1038/nrc.2017.79 PMC6000833

[9] Wilhelm SM, Carter C, Tang L, et al. BAY 43-9006 exhibits broad spectrum oral antitumor activity and targets the RAF/MEK/ERK pathway and receptor tyrosine kinases involved in tumor progression and angiogenesis. Cancer Res 2004;64:7099–109. 15466206 10.1158/0008-5472.CAN-04-1443

[10] Lapierre J-M, Namdev ND, Ashwell MA, et al. RAF inhibitors and their uses. PCT Pat Appl WO 2007/123892.

[11] Abdel-Maksoud MS, Kim M-R, El-Gamal MI, et al. Design, synthesis, in vitro antiproliferative evaluation, and kinase inhibitory effects of a new series of imidazo[2,1-b]thiazole derivatives. Eur J Med Chem 2015;95:453–63. 25841200 10.1016/j.ejmech.2015.03.065

[12] Ammar UM, Abdel-Maksoud MS, Ali EMH, et al. Structural optimization of imidazothiazole derivatives affords a new promising series as B-Raf V600E inhibitors; synthesis, *in vitro* assay and *in silico* screening. Bioorg Chem 2020;100:103967 32470760 10.1016/j.bioorg.2020.103967

[13] Park J-H, Oh C-H. Synthesis of new 6-(4-fluorophenyl)-5-(2-substituted pyrimidin-4-yl)imidazo[2,1-b]thiazole derivatives and their antiproliferative activity against melanoma cell line. Bull Korean Chem Soc 2010;31:2854–60.

[14] Park J-H, El-Gamal MI, Lee YS, et al. New imidazo[2,1-*b*]thiazole derivatives: Synthesis, *in vitro* anticancer evaluation, and *in silico* studies. Eur J Med Chem 2011;46:5769–77. 22033063 10.1016/j.ejmech.2011.08.024

[15] Abdel-Maksoud MS, El-Gamal MI, Gamal El-Din MM, et al. Broad-spectrum antiproliferative activity of a series of 6-(4-fluorophenyl)-5-(2-substituted pyrimidin-4-yl)imidazo[2,1-b]thiazole derivatives. Med Chem Res 2016;25:824–33.

[16] El-Gamal MI, Abdel-Maksoud MS, Gamal El-Din MM, et al. Synthesis, *in vitro* antiproliferative and antiinflammatory activities, and kinase inhibitory effects of new 1,3,4-triarylpyrazole derivatives. Anticancer Agents Med Chem 2017;17:75–84. 27334850

[17] Pedretti A, Villa L, Vistoli G. Atom-type description language: a universal language to recognize atom types implemented in the VEGA program. Theor Chem Acc 2003;109:229–32.

[18] Pedretti A, Villa L, Vistoli G. VEGA – An open platform to develop chemo-bio-informatics applications, using plug-in architecture and script programming. J Comput Aid Mol Des 2004;18:167–73. 10.1023/b:jcam.0000035186.90683.f2 15368917

[19] Trott O, Olson AJ. AutoDock Vina: improving the speed and accuracy of docking with a new scoring function, efficient optimization, and multithreading. J Comput Chem 2010;31:455–61. 19499576 10.1002/jcc.21334 PMC3041641

[20] Baroni M, Cruciani G, Sciabola S, et al. A common reference framework for analyzing/comparing proteins and ligands. Fingerprints for Ligands and Proteins (FLAP): theory and application. J Chem Inf Model 2007;47:279–94. 17381166 10.1021/ci600253e

[21] Cross S, Baroni M, Goracci L, et al. GRID-Based Three-Dimensional Pharmacophores I: FLAPpharm, a Novel Approach for Pharmacophore Elucidation. J Chem Inf Model 2012;52:2587–98. 22970894 10.1021/ci300153d

[22] Milletti F, Storchi L, Sforna G, et al. New and original p*K*_a_ prediction method using grid molecular interaction fields. J Chem Inf Model 2007;47:2172–81. 17910431 10.1021/ci700018y

[23] Crivori P, Cruciani G, Carrupt P-A, et al. Predicting blood-brain barrier permeation from three-dimensional molecular structure. J Med Chem 2000;43:2204–16. 10841799 10.1021/jm990968+

[24] Cruciani G, Crivori P, Carrupt P-A, et al. Molecular fields in quantitative structure-permeation relationships: the VolSurf approach. Theochem 2000;503:17–30.

[25] Gramatica P, Chirico N, Papa N, et al. QSARINS: a new software for the development, analysis, and validation of QSAR MLR models. J Comput Chem 2013;34:2121–32.

[26] Gramatica P, Cassani S, Chirico N. QSARINS-chem: Insubria datasets and new QSAR/QSPR models for environmental pollutants in QSARINS. J Comput Chem 2014;35:1036–44. 24599647 10.1002/jcc.23576

[27] Ashwell M, Tandon M, Lapierre J-M. Synthesis of imidazooxazole and imidazothiazole inhibitors of p38 map kinase. PCT Patent Application WO 2006044869, 2006.

[28] Tully DC, Liu H, Chatterjee AK, et al. Synthesis and SAR of arylaminoethyl amides as noncovalent inhibitors of cathepsin S: P3 cyclic ethers. Bioorg Med Chem Lett 2006;16:5112–7. 16876402 10.1016/j.bmcl.2006.07.033

[29] Townsend CA, Basak A. Experiments and speculations on the role of oxidative cyclization chemistry in natural product biosynthesis. Tetrahedron 1991;47:2591–602.

[30] Sumimoto H, Imabayashi F, Iwata T, et al. The BRAF-MAPK signaling pathway is essential for cancer-immune evasion in human melanoma cells. J Exp Med 2006;203:1651–6. 16801397 10.1084/jem.20051848 PMC2118331

[31] Carosati E, Sciabola S, Cruciani G. Hydrogen bonding interactions of covalently bonded fluorine atoms: From crystallographic data to a new angular function in the GRID force field. J Med Chem 2004;47:5114–25. 15456255 10.1021/jm0498349

